# Glycoproteome Profiling of Human Serum for Hepatocellular Carcinoma Biomarker Discovery

**DOI:** 10.1021/acs.jproteome.6c00258

**Published:** 2026-06-17

**Authors:** Muhammad Salman Sajid, Rency S. Varghese, Shafaq Saleem, Xinran Zhang, Alexander Kroemer, Habtom W. Ressom

**Affiliations:** † Department of Oncology, Lombardi Comprehensive Cancer Center, 12231 Georgetown University Medical Center, Washington, District of Columbia 20057, United States; ‡ MedStar Georgetown Transplant Institute, MedStar Georgetown University Hospital, and the Center for Translational Transplant Medicine, Georgetown University Medical Center, Washington, District of Columbia 20057, United States

**Keywords:** liver cancer, glycoproteomics, biomarker, human serum, nano-LC-MS/MS

## Abstract

Hepatocellular carcinoma (HCC) typically develops in a cirrhotic background, where elevated serum α-fetoprotein (AFP), a commonly used HCC biomarker, performs inconsistently. We implemented an integrated serum intact glycoproteomics strategy that profiles N-linked and O-linked glycopeptides in parallel. This strategy couples prevalence-aware filtering with multialgorithm feature selection to prioritize glycoform-resolved markers distinguishing HCC from cirrhosis. Serum from HCC (*n* = 20) and cirrhosis (*n* = 20) underwent high-abundance protein depletion, proteolysis, glycopeptide enrichment, and high-resolution liquid chromatography–mass spectrometry/MS (LC–MS/MS). Intact glycopeptides were filtered to a high-confidence quantitative matrix requiring ≥70% feature presence, with statistically significant low-prevalence features recovered by chi-square testing and incorporated into the final modeling pool. Candidate prioritization integrated SelectKBest, SVM-RFE, Elastic Net, Transformer-RFE, and Random Forest, with discrimination assessed by ROC analysis. This workflow delivered broad N/O glycoproteome coverage and a candidate of 11 intact glycopeptide features spanning both glycosylation classes. Multiple candidates exhibited pronounced group-associated detectability and/or significant abundance differences, and prioritized markers exceeded clinically measured AFP in univariate ROC comparisons within this cohort. Network-level interpretation (PPI and IPA) revealed an immune/complement-centered module and enrichment of pathways encompassing the complement system, neutrophil degranulation, O-linked glycosylation, sphingolipid metabolism, and nuclear receptor signaling (LXR/RXR activation and androgen signaling).

## Graphical abstract

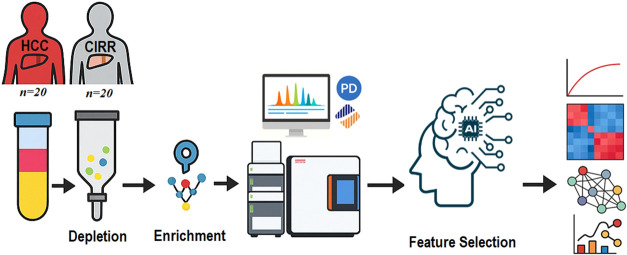

## Introduction

Hepatocellular carcinoma (HCC) is the most common form of primary liver cancer and a leading cause of cancer-related mortality worldwide.^1^ Most patients develop HCC on a background of chronic liver disease, particularly cirrhosis, and the clinical challenge lies not only in detecting HCC early but also in distinguishing HCC from cirrhosis, a condition with overlapping biochemical and inflammatory signatures.^2–4^ Current surveillance biomarkers such as α-fetoprotein (AFP) lack adequate sensitivity and specificity, especially in patients with cirrhosis,^5,6^ highlighting a persistent need for improved molecular markers that reflect the complex biology of liver injury and malignant transformation.^7,8^ Because the liver is the primary source of many circulating proteins, changes in hepatocyte biology^9^ and tumor microenvironment are directly reflected in serum protein composition and post-translational modifications, making serum an ideal matrix for biomarker discovery.^10,11^

Protein glycosylation plays an especially important role in liver disease.^12^ Dysregulated glycosylation influences protein folding, secretion, immune signaling, cell–cell interactions, and malignant progression. Numerous studies have shown that altered fucosylation, sialylation, branching, and glycan occupancy occur early in chronic liver disease and become more pronounced during the transition from cirrhosis to HCC.^13,14^ These disease-associated glycosylation changes are often site-specific, affecting particular glycoforms on proteins such as α-2-HS-glycoprotein (AHSG), complement components, interalpha-trypsin inhibitors, immunoglobulins, and other liver-derived proteins.^15^ This makes the serum glycoproteome a rich source of diagnostic and mechanistic information for HCC.

Traditional glycomics and deglycosylated proteomics approaches, however, have important limitations: they provide global glycan information but lose the identity of the exact peptide sequence and glycosylation site, making it difficult to determine how disease biology reshapes glycosylation at the protein- and site-specific level.^16^ In contrast, intact glycopeptide analysis using liquid chromatography–mass spectrometry/MS (LC–MS/MS) has emerged as a powerful strategy to map the precise peptide backbone, glycosylation site, and glycan composition in a single measurement. This site-specific readout enables direct quantification of glycoform microheterogeneity information that is essential for understanding how glycosylation shifts during HCC development.^17^ Despite these advantages, intact glycopeptide profiling in human serum remains analytically challenging due to low stoichiometry, extensive glycan microheterogeneity, the wide dynamic range of serum proteins, and the fragile nature of glycan–peptide linkages during fragmentation.^18^

Advances in enrichment methods, nanoLC separations, and dedicated glycoproteomics search engines have greatly expanded the coverage of intact N-linked glycopeptides in serum.^19^ However, O-linked glycopeptides remain difficult to analyze because they lack a consensus motif and display even greater structural diversity, resulting in fewer studies that profile N- and O-glycopeptides together.^20^ Additionally, intact glycopeptide data sets are often high-dimensional but sparse, with substantial missing values and variable detection frequencies across samples. These properties complicate traditional univariate statistical analyses, which can overlook subtle but biologically important differences.^21^ Thus, while intact glycopeptidomics provides high-resolution molecular information, advanced approaches such as machine learning (ML) are desired to fully leverage its potential in biomarker discovery.

Machine learning has recently gained traction in proteomics and glycomics as a powerful tool for capturing multivariate patterns that distinguish disease states. It can integrate subtle covariations across hundreds of features, identify combinations of markers with better discriminatory performance than individual analytes, and handle complex data structures that traditional statistics may miss.^22^ In liver cancer research, ML has been applied to transcriptomics, imaging, metabolomics, and protein-level glycoproteomics, demonstrating its ability to uncover meaningful molecular signatures.^23–25^ Yet, ML-driven biomarker discovery at the level of intact glycopeptides, where the richest site-specific glycan information resides, remains largely unexplored.

In this study, we aimed to identify clinically meaningful glycopeptide markers for HCC by combining comprehensive serum glycoproteomics with machine learning–based analysis. We generated quantitative profiles of intact N-linked and O-linked glycopeptides from patients with HCC and CIRR. We analyzed them using an integrated framework designed to handle the complexity and sparsity of serum glycoproteomics data. Overall, following the FDA-NIH BEST Resource terminology, this study defines a discovery-stage set of candidate intact glycopeptide markers intended to distinguish HCC from cirrhosis, rather than prognostic, predictive, or monitoring biomarkers.

## Materials and Methods

### Materials and Chemicals

High-abundance serum proteins were depleted using Pierce Top 12 Abundant Protein Depletion Spin Columns (Thermo Fisher Scientific, Cat. No. 85165). Protein digestion was carried out by filter-aided sample preparation (FASP) using Amicon Ultra-0.5 centrifugal filters (30 kDa MWCO) (MilliporeSigma, Cat. No. UFC501024) and sequencing-grade modified trypsin (Promega, Cat. No. V5111). Glycopeptides were enriched using Oasis MAX 1 cc (10 mg) cartridges (Waters Corporation, Cat. No. 186000366), followed by peptide desalting on Oasis HLB 1 cc (10 mg) cartridges (Waters Corporation, Cat. No. 186000383). All solvents and reagents were of the highest purity available. Acetonitrile (LC–MS grade; Cat. No. A955), formic acid (LC–MS grade; ≥99%; Cat. No. 85178), trifluoroacetic acid (≥99%; Cat. No. 28903), dithiothreitol (≥99%; Cat. No. R0861), iodoacetamide (≥99%; Cat. No. I1149), and ammonium bicarbonate (≥99.5%; Cat. No. A6141) were obtained from Thermo Fisher Scientific or Sigma-Aldrich. Ultrapure water (18.2 MΩ·cm) was generated using a Milli-Q purification system (Millipore). LC–MS/MS analyses were performed on a Q Exactive Orbitrap mass spectrometer coupled to an Ultimate 3000 nanoLC system (Thermo Fisher Scientific). Glycopeptide identification and label-free quantification were conducted using Proteome Discoverer (v3.0) integrated with the Byonic node (Protein Metrics Inc.).

### Experimental Design

To systematically discover glycopeptide-based markers for HCC, we implemented an integrated experimental and computational workflow, summarized in Figure 1. The study was designed to comprehensively investigate both N-linked and O-linked glycopeptides in human serum, with the primary objective of biomarker discovery. Serum samples from patients with HCC (*n* = 20) and cirrhosis (*n* = 20) were subjected to depletion of high-abundance proteins, followed by filter-aided sample preparation and targeted enrichment of glycopeptides. The enriched fractions were analyzed by high-resolution LC–MS/MS on a Q Exactive Orbitrap platform, allowing for the site-specific identification and quantification of intact glycopeptides. Glycopeptide identification and annotation were performed using the Byonic search engine within Proteome Discoverer, allowing simultaneous determination of peptide backbone, glycosylation site, and glycan composition. To ensure high-confidence measurements suitable for downstream biomarker discovery, stringent quality control and filtering criteria were applied, including a peptide-level false discovery rate ≤1% and a minimum feature presence threshold of ≥70% across samples. Both N-linked and O-linked glycopeptide data sets were processed in parallel to preserve complementary information arising from these two major glycosylation classes. With the explicit goal of identifying discriminative markers for HCC, the filtered data sets were subjected to an integrated feature selection framework that combines conventional statistical testing with multiple machine learning approaches. This framework incorporated SelectKBest, support vector machine recursive feature elimination (SVM-RFE), transformer-RFE, and random forest algorithms to identify glycopeptide features most strongly associated with disease state. Model performance was evaluated using receiver operating characteristic (ROC) analysis and area under the curve (AUC) metrics, while biological interpretation of candidate markers was supported by expression profiling, protein–protein interaction analysis, and functional pathway enrichment. Together, this workflow provided a robust and reproducible platform for the large-scale interrogation of intact N-linked and O-linked glycopeptides in human serum, establishing the foundation for the subsequent identification of glycosylation-based markers that distinguish HCC from cirrhosis.

**Figure 1:**
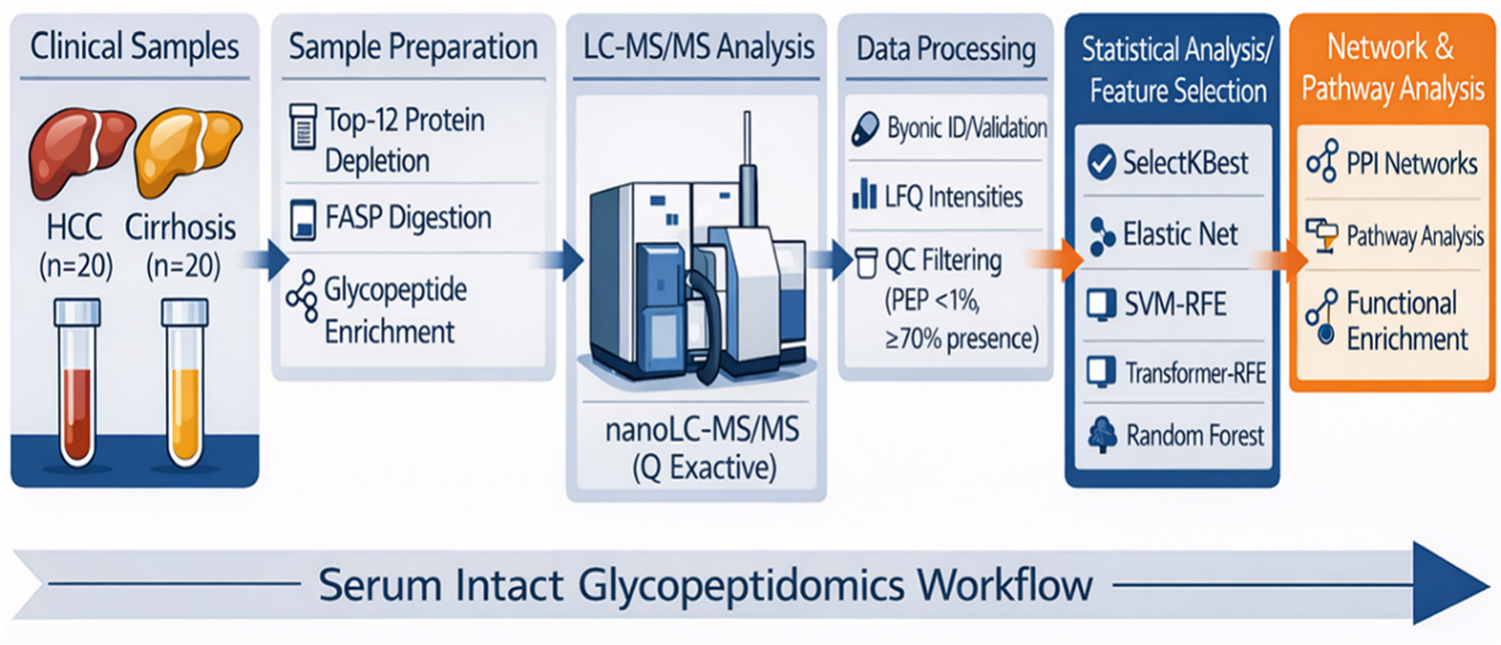
Overview of the workflow for the untargeted serum intact glycoproteomics study.

### Study Cohort

Serum samples from 20 HCC cases and 20 liver cirrhosis patients were recruited at MedStar Georgetown University Hospital. All participants filled out HIPAA authorization forms and provided their informed consent. The characteristics of the subjects whose serum samples were analyzed are shown in Table 1. Every HCC patient in this study had a diagnosis of liver cirrhosis. Well-established criteria for diagnostic imaging and/or histology were used to diagnose cases with HCC. The tumor–node–metastasis (TNM) classification system was used to determine the clinical stages of HCC.

**Table 1:** Characteristics of the Patients Whose Serum Samples Were Analyzed^a^

		HCC (*n* = 20)	CIRR (*n* = 20)	*p*-value
age	mean (SD)	59 (6)	57 (6)	0.487
sex	male	60%	65%	1
race	AA	50%	40%	0.5231
	EA	50%	60%	
HCV serology	HCV Ab+	80%	75%	0.6948
HBV serology	anti HBC+	45%	40%	0.7431
	HBsAg+	5%	0	1
smoking	current	25%	25%	1
	former	55%	50%	
alcohol	current	25%	20%	0.6851
	former	55%	60%	
MELD*	median (IQR)	10.5 (5.2)	13.5 (9.2)	0.0475
AFP	median (IQR)	29.1 (60.8)	7 (35.1)	0.113
HCC stage	stage I	30%		
	stage II	65%		
	stage III	5%		

a Characteristics with statistically significantly different values (*p* < 0.05) between the two patient groups are marked with an asterisk. AFP was measured in ng/mL.

### Sample Preparation

From each sample, 10 μL of serum was processed to remove the most abundant proteins using High-Select Top14 Abundant Protein Depletion Mini Spin Columns (Thermo Fisher Scientific, Cat. No. A36370) according to the manufacturer’s protocol. Flow-through fractions containing low-abundance proteins were buffer-exchanged and concentrated using Amicon Ultra-0.5 centrifugal filters, 10 kDa MWCO (Millipore, Cat. No. UFC501096). Protein concentration was measured with the Pierce BCA Protein Assay Kit (Thermo Fisher Scientific, Cat. No. 23225).

The depleted samples were subjected to our previously optimized filter-aided sample preparation (FASP) with minor changes.^26^ Samples were solubilized in 6 M urea/100 mM ABC, pH 8.0, on 10 kDa MWCO filters; reduced with 10 mM dithiothreitol (DTT; Sigma, Cat. No. D0632) for 45 min at 37 °C; and alkylated with 25 mM iodoacetamide (IAA; Sigma, Cat. No. I6125) for 30 min at room temperature in the dark. After buffer exchange with 50 mM ammonium bicarbonate (Sigma, Cat. No. 09830), proteins were digested overnight at 37 °C with sequencing-grade modified trypsin (Promega, Cat. No. V5111) at a 1:50 enzyme/substrate ratio. Peptides were recovered by centrifugation and rinsed from the filter with 50 mM ammonium bicarbonate.

The resulting digests were enriched for N- and O-linked glycopeptides using Oasis MAX HILIC cartridges (Waters, Cat. No. 186008051). Cartridges were conditioned with 100% acetonitrile (ACN; Thermo Fisher Scientific, Cat. No. A955–4) and equilibrated with 95% ACN/1% trifluoroacetic acid (TFA; Thermo Fisher Scientific, Cat. No. 85183). Peptides dissolved in the same buffer were loaded onto the cartridges and washed to remove nonglycosylated peptides, and glycopeptides were eluted with 0.1% formic acid (FA; Thermo Fisher Scientific, Cat. No. 28905). Eluted fractions were dried in a vacuum concentrator and desalted before injection.

### LC MS/MS Data Acquisition

For LC–MS/MS analysis, 1 μg of enriched peptides was resuspended in 0.1% FA and injected onto an Ultimate 3000 RSLCnano system (Thermo Fisher Scientific) coupled to a Q Exactive Quadrupole-Orbitrap mass spectrometer (Thermo Fisher Scientific). Samples were first loaded onto a 100 μm × 2 cm C18 trap column (Thermo Fisher Scientific, Cat. No. 164564) and then separated on a 75 μm × 25 cm C18 analytical column with a 2 μm particle size (Thermo Fisher Scientific, Cat. No. ES802). Peptides were resolved using a 90 min linear gradient from 5% to 35% ACN in 0.1% FA at 300 nL/min. The Q Exactive was operated in data-dependent acquisition (DDA) mode with full MS scans at a resolution of 70,000 (*m*/*z* 200, scan range 350–1600 *m*/*z*), followed by HCD fragmentation of the top 15 precursor ions at normalized collision energy (NCE) 28. MS/MS spectra were acquired at 17,500 resolution with AGC targets of 3e^6^ (MS) and 1e^5^ (MS/MS), a maximum injection time of 100 ms, and dynamic exclusion of 30s. The mass spectrometry proteomics data have been deposited to the ProteomeXchange Consortium via the PRIDE partner repository with the data set identifier PXD07537.

### Data Processing

Raw LC-MS/MS data were processed using Proteome Discoverer v3.0 (Thermo Fisher Scientific) with Byonic (Protein Metrics) as a search node. Searches were performed against the UniProt human reference proteome database with trypsin specificity (maximum two missed cleavages). Carbamidomethylation (Cys) was set as a fixed modification, while oxidation (Met) and N-/O-glycan modifications from the Byonic database were specified as variables. Precursor and fragment mass tolerances were 10 and 20 ppm, respectively. Peptide-spectrum matches were filtered to a 1% FDR. Label-free quantification (LFQ) was carried out in Proteome Discoverer consensus workflows based on precursor intensities, with normalization across samples applied to enable relative comparison of glycopeptide abundances between HCC and CIRR groups. Principal component analysis (PCA) performed using LFQ intensities of all glycopeptides was used to identify any potential outliers. Peptides detected in less than 70% of at least one patient group were left aside for subsequent analysis based on absent and present calls via Pearson’s chi-squared test. Missing features were imputed as half of the lowest observed value for that feature across all samples.

### Feature Selection

Various statistical and machine learning methods including SelectKBest, Elastic Net, Support Vector Machine-Recursive Feature Elimination (SVM-RFE), Transformer-RFE, and Random Forest (RF) were applied to select a panel of glycopeptides that distinguishes HCC cases from cirrhotic patients. Features consistently selected across multiple methods were prioritized as the most robust candidates for the final panel. We applied these methods previously in other omics studies.^25,27^ Briefly, SelectKBest is a scikit-learn filter method that uses a univariate F-test to score features and keeps the top K.^28^ Elastic Net combines L1 (Lasso) and L2 (Ridge) penalties, shrinking coefficients and effectively selecting features by driving some to zero.^29^ SVM-RFE iteratively trains an SVM, ranks features by weight magnitude, and removes the least important until the target set remains.^30^ Transformer-RFE follows the same iterative idea but uses a lightweight cross-attention transformer and SHAP scores to drop the least important features with retraining each round.^27^ Random forest is an ensemble of bootstrapped decision trees; feature importance can be ranked using SHAP based on each feature’s contribution to predictions.^31^

### Pathway Analysis

Differentially expressed glycoproteins were further evaluated for pathway enrichment and functional correlations using Ingenuity Pathway Analysis (IPA) software (QIAGEN Inc., Germantown, MD, USA). Protein identifiers and corresponding expression changes were uploaded into IPA, and Core Analysis was performed to identify significantly enriched canonical pathways, upstream regulators, and disease/function annotations based on the IPA knowledge base. Pathways were prioritized using the *p*-value from Fisher’s exact test.

## Results

Human serum samples from hepatocellular carcinoma (HCC, *n* = 20) and cirrhosis (CIRR, *n* = 20) subjects were included in this study. Clinical and demographic characteristics are summarized in Table 1. Most baseline variables were comparable between groups; however, MELD score differed between HCC and CIRR (*p* ≈ 0.0475) (Table 1). All samples were processed using the same depletion, digestion, desalting, and glycopeptide enrichment workflow, followed by LC–MS analysis and glycopeptide annotation. Principal component analysis (PCA) was used to evaluate whether N-linked and O-linked intact glycopeptide profiles capture group-associated structure between HCC (*n* = 20) and cirrhosis (CIRR; *n* = 20). After applying the study-wide filtering criteria (high-confidence Byonic annotations) followed by log_2_ transformation and normalization, PCA was performed separately for the N-linked and O-linked feature matrices. In both data sets, the PCA score plots (Figures S1 and S2) showed partial separation of HCC and CIRR samples along the first two principal components. For the N-linked data set, PC1 and PC2 explained 14.7% and 9.0% of the total variance, respectively, whereas for the O-linked data set, PC1 and PC2 explained 11.6% and 10.2% of the variance, respectively.

### Quality Control and Analytical Performance

Instrument performance and data reproducibility were monitored throughout the study using repeated injections of a standard HeLa digest (QC) sample, with 200 ng injected per run. Nine QC injections were acquired before, during, and after analysis of the clinical cohort, and the resulting performance metrics are summarized in Figure 2. Protein identifications remained stable across the QC sequence (Figure 2a), with approximately 3200–3400 proteins identified per injection and no evidence of systematic drift over time. Peptide identifications showed a similar pattern (Figure 2b), with roughly 14,000–17,000 peptides detected per run, reflecting consistent sampling depth and instrument sensitivity across the acquisition period. Chromatographic reproducibility was evaluated by retention time coefficient of variation (RT CV) analysis (Figure 2c). The median RT CV was below 1%, and nearly all peptide features remained well under the 2% acceptance threshold, indicating robust chromatographic stability throughout the experiment. Quantitative precision was assessed using the distribution of peptide-level intensity CVs derived from normalized abundances (Figure 2d). Most peptide features exhibited CVs below 30%, with a gradually decreasing tail toward higher values, consistent with expected behavior for low-abundance features in complex serum-derived samples. Collectively, these QC measurements demonstrate that the LC–MS/MS platform operated with stable identification performance, reproducible chromatography, and acceptable quantitative precision over the full acquisition window, providing a reliable foundation for subsequent comparative glycoproteomic analyses.

**Figure 2:**
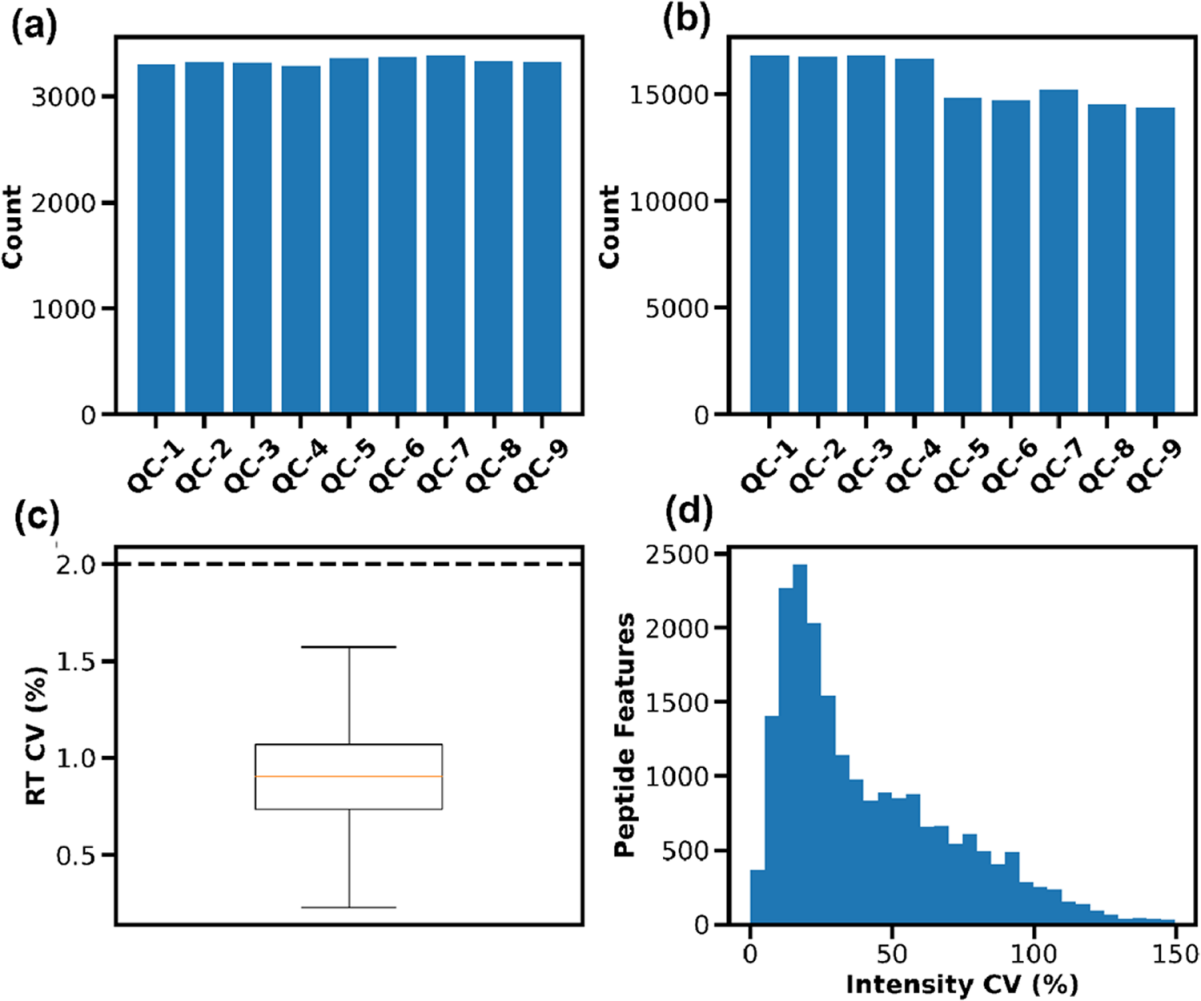
Quality control assessment of LC–MS/MS performance: (a) Proteins identified across nine quality-control (QC) injections, (b) Peptides identified across QC injections, (c) Retention time (RT) reproducibility shown as a boxplot of RT coefficients of variation (CV%), with the dashed line indicating a 2% threshold, and (d) Distribution of peptide-level intensity CVs calculated from normalized abundances across QC injections.

Using the integrated workflow, we generated paired N-linked and O-linked intact glycoproteomics data sets from the depleted serum cohort, providing broad glycoproteome coverage (Figure 3). The N-linked data set comprised 1050 intact glycopeptides mapping to 108 glycoproteins and 721 unique glycans (Table S1), whereas the O-linked data set comprised 411 intact glycopeptides mapping to 103 glycoproteins and 324 unique glycans (Figure 3a and Table S2). In this study, each intact glycopeptide was treated as a distinct glycoform (peptide sequence + glycan composition), yielding 1050 N-linked glycoforms (200 N-glycosylation sites) and 410 O-linked glycoforms with 43 O-glycosylation sites (Figure 3a). Comparison across glycosylation types demonstrated partial complementarity in glycoprotein coverage, with 36 glycoproteins shared between the N-linked and O-linked data sets and additional proteins detected uniquely in each data set (73 N-only and 68 O-only glycoproteins; Figure 3b). Glycoform diversity per protein showed a broader distribution for N-linked glycoproteins, including proteins with substantially higher numbers of glycoforms, whereas O-linked glycoproteins exhibited a narrower distribution with fewer glycoforms per protein overall (Figure 3c). Reproducibility was assessed at the protein level by examining coefficient of variation (CV) distributions across QC and clinical strata, yielding median protein CVs of 0.64 (QC), 0.76 (N-CIRR), 0.74 (N-HCC), 0.63 (O-HCC), and 0.60 (O–CIRR) (Figure 3d). Abundance rank plots further indicated that a limited set of high-abundance glycoproteins accounted for a substantial fraction of total signal in each data set, with the top contributors explaining 69.3% of total protein abundance in the N-linked data and 73.0% in the O-linked data (Figure 3(e,f)). At the glycoprotein level, extensive overlap was observed between disease groups: 162 glycoproteins were detected in HCC and 173 in cirrhosis, with 162 shared between the two groups (HCC ∩ CIRR), 0 HCC-only, and 11 cirrhosis-only glycoproteins under the applied presence criterion; restricting to the N-linked subset, all 108 N-linked glycoproteins were shared across HCC and cirrhosis. In contrast, group-associated differences were more apparent at the intact glycopeptide level, where overlap analysis identified 504 shared glycopeptides, 215 cirrhosis-specific, and 39 HCC-specific glycopeptides based on the applied presence criteria, consistent with disease-associated variation emerging at the glycoform/site-resolved level.

**Figure 3:**
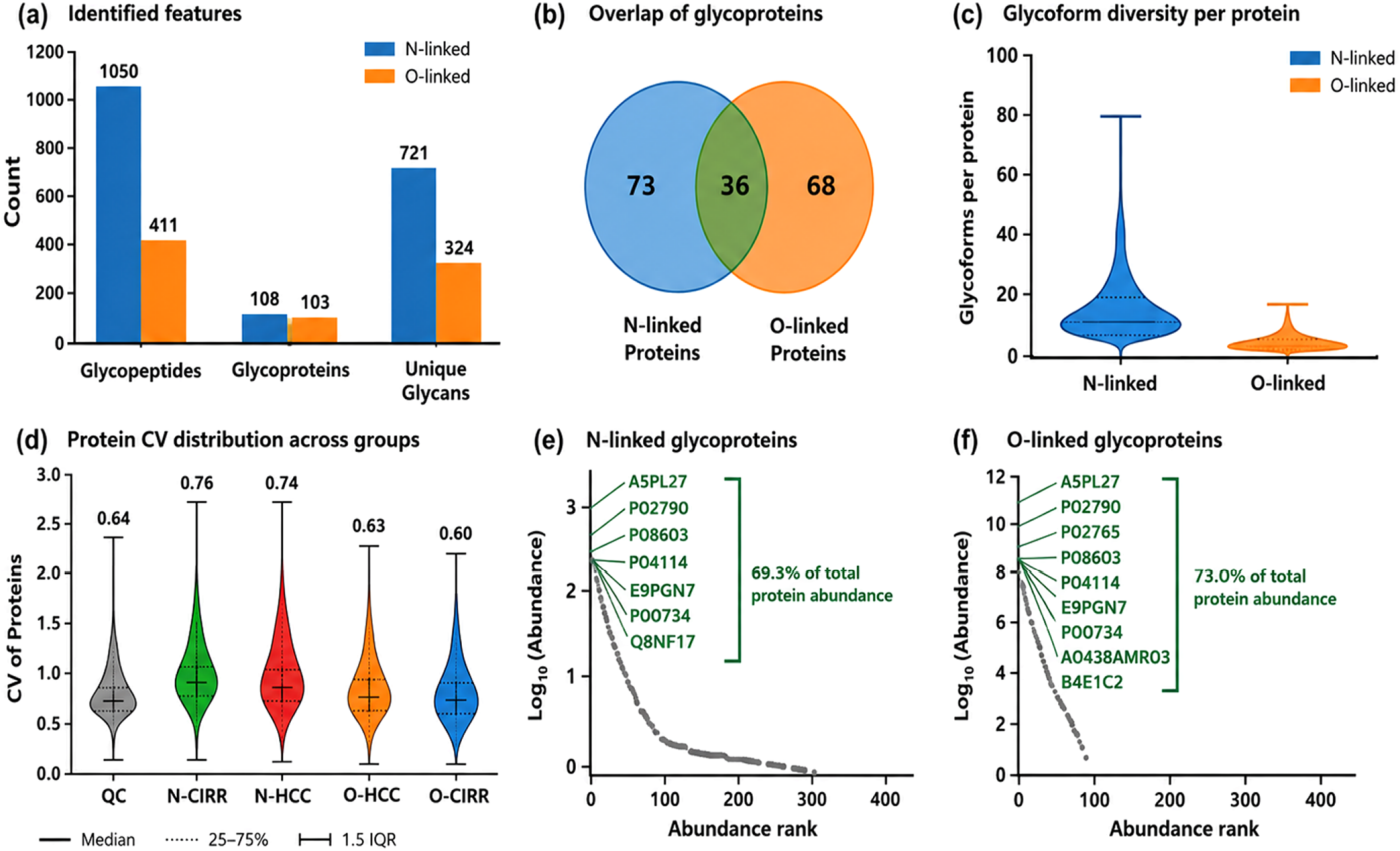
Overview of N- and O-linked serum glycoproteome coverage and reproducibility. (a) Identified glycopeptides, glycoproteins, and unique glycans. (b) Overlap of N- and O-linked glycoproteins. (c) Glycoform diversity per protein. (d) Protein-level CV distributions across QC, N-CIRR, N-HCC, O–CIRR, and O-HCC groups. (e–f) Abundance rank plots for N-linked and O-linked glycoproteins, respectively.

Glycopeptide features were filtered to construct a primary quantitative matrix by requiring a minimum presence of ≥70% across samples; the remaining lower-prevalence features (<30% presence) were evaluated using chi-square testing, and significant candidates from both N-linked and O-linked data sets were combined with the ≥70% matrix to form the final feature pool used for modeling. Feature prioritization was performed separately and combined for N-linked and O-linked glycopeptides using multiple approaches (SelectKBest, SVM-RFE, Elastic Net, Transformer-RFE, and Random Forest), and glycopeptides ranked among the top features by more than one method were retained as robust candidates (Tables S3–S5).

In the N-linked data set (Table S3), repeatedly prioritized candidates spanned a range of detection patterns, including strong prevalence differences such as attractin (*ARTN*) (VFHIHNESWVLLTPK; HexNAc_2_Hex_4_) detected in 19/20 cirrhosis samples and 0/20 HCC samples and FLJ00385 protein (*FLJ00385*) (EEQFNSTFR; HexNAc_4_Hex_3_) detected in 10/20 cirrhosis and 0/20 HCC, as well as candidates observed predominantly in HCC such as hemopexin (*HPX*) (ALPQPQNVTSLLGCTH; HexNAc_4_Hex_5_) detected in 19/20 HCC versus 10/20 cirrhosis and afamin **(** *AFM* **)** (FNETTEK; HexNAc_5_Hex_6_FucNeuAc_2_) detected in 13/20 HCC versus 3/20 cirrhosis; additional N-linked glycopeptides detected broadly in both groups were repeatedly prioritized, including properdin (*CFP*) (NVTFWGR; HexNAc_4_Hex_5_FucNeuAc) and sex hormone-binding globulin (*SHBG*) (SHEIWTHSCPQSPGNGTDASH; HexNAc_4_Hex_5_NeuAc). Classification performance was evaluated by ROC analysis, yielding AUCs of 0.990 (SelectKBest), 1.000 (SVM-RFE), 0.928 (Elastic Net), 0.968 (Transformer-RFE), and 1.000 (RF).

In the O-linked data set (Table S4), multiple candidates showed pronounced group-associated detection differences, including chemokine (C-X3-C motif) ligand 1 variant (AQDGGPVGTELFR; HexNAcHexNeuAc_2_) detected in 15/20 cirrhosis and 1/20 HCC and IGH c1218 heavy IGHV3–48 IGHD2–2 IGHJ6 (EVQMVDSGGGLVQPGGSLR; HexNAcHexNeuAc) detected in 20/20 cirrhosis and 15/20 HCC, together with additional prioritized features such as IG c657 heavy IGHV3–7 IGHD5–12 IGHJ4 (QDGSEK; HexNAc_2_HexFucNeuAc) detected in 20/20 cirrhosis versus 2/20 HCC and serpin family A member 1 (SERPINA1) (DTEEEDFHVDQATTVK; HexNAc_2_Hex_2_NeuAc) was detected in 10/20 HCC versus 1/20 cirrhosis; features detected in all samples of both groups (e.g., IG c657 heavy IGHV3–7 IGHD5–12 IGHJ4 and kininogen-1) were also selected by more than one method. AUCs were 0.993 (SelectKBest), 0.998 (SVM-RFE), 0.993 (Elastic Net), 0.998 (Transformer-RFE), and 0.998 (RF).

When the N-linked and O-linked matrices were combined and the same five approaches were applied to identify the top five discriminative glycopeptides per method (Table S5), the resulting candidate sets included prevalence-skewed features such as *ARTN* (19/20 cirrhosis; 0/20 HCC), complement component C8 α chain (*C8A*) (20/20; 2/20), prosaposin (*PSAP*) (17/20; 2/20), cDNA FLJ51742, highly similar to interalpha-trypsin inhibitor heavy (19/20; 1/20), and IG c657 heavy IGHV3–7 IGHD5–12 IGHJ4 (20/20; 2/20), alongside features detected broadly in both groups (e.g., IG c657 heavy IGHV3–7 IGHD5–12 IGHJ4 and kininogen-1, each 20/20 in both groups), with accuracies of 0.925 (SelectKBest), 0.925 (SVM-RFE), 0.975 (Elastic Net), 0.900 (Transformer-RFE), and 0.875 (Random Forest) and corresponding AUC values of 0.993, 1.000, 0.995, 0.908, and 1.000, respectively (Table S5).

Based on frequent prioritization across the above three workflows, a candidate set of 11 intact glycopeptides was finalized; each candidate was selected by more than one feature selection method (Table 2). These include prevalence-skewed features such as *ARTN* (0/20 HCC vs 19/20 CIRR) and IG c657_heavy_IGHV3–7_IGHD5–12_IGHJ4 (2/20 vs 20/20) and chemokine (C-X3-C motif) ligand 1 variant (1/20 vs 15/20), as well as broadly detected candidates with significant abundance differences, including *CFP* (20/20 and 20/20); IGH c3277_heavy_IGHV3–23_IGHD3–22_IGHJ4 (IGH c3277) (20/20 and 20/20); and IGH c1218 heavy IGHV3–48 IGHD2–2 IGHJ6 (IGH c1218) (15/20 vs 20/20). Table 2 lists the 11 intact glycopeptides along with the number of samples in which they were detected (HCC vs CIRR), the fold change (FC) values, and MELD-adjusted *p*-values obtained using a logistic regression method following imputation of each missing value as half of the lowest observed value for that feature across all samples.

**Table 2:** Selected 11 Intact Glycopeptides^a^

				detected in			
gene name	sequence	modifications	MH+ [Da]	HCC	CIRR	MELD-adjusted *p*-value	FC
*ATRN*	VFHIHNESWVLLTPK	HexNAc_2_Hex_4_	2874.35	0	19	2.2 × 10^–08^	–24.4
*AHSG*	VCQDCPLLAPLNDTR	HexNAc_5_Hex_6_Fuc_2_NeuAc_2_	4633.86	11	20	1.4 × 10^–08^	–131.4
*C8A*	GGSSGWSGGLAQNR	HexNAc_4_Hex_5_NeuAc_2_	3538.39	2	20	8.3 × 10^–10^	–92.8
*CFP*	NVTFWGR	HexNAc_4_Hex_5_FucNeuAc	2939.18	20	20	3.9 × 10^–09^	–8.73
*SHBG*	SHEIWTHSCPQSPGNGTDASH	HexNAc_4_Hex_5_NeuAc	4218.64	3	19	2.5 × 10^–08^	–15.3
*ITIH4*	TEETTMTTQTPAPIQAPSAILPLPGQSVER	HexNAc_4_Hex	4138.98	1	19	7.8 × 10^–10^	–97.5
*PSAP*	TNSTFVQALVEHVK	HexNAc_2_Hex_2_Fuc	2449.16	2	17	3.7 × 10^–05^	–5.5
*IGH c3277*	EQLSQSGGGLVQPGGSLR	HexNAc_3_	2379.15	20	20	0.001	–1.9
*IGH c1218*	EVQMVDSGGGLVQPGGSLR	HexNAc Hex NeuAc	2542.17	15	20	0.033	1.6
*CX3CL1*	AQDGGPVGTELFR	HexNAc Hex NeuAc_2_	2293.99	1	15	1.2 × 10^–04^	–7.4
*IG c657*	QDGSEK	HexNAc_2_Hex Fuc NeuAc	1668.65	2	20	4.5 × 10^–08^	–108.3

a For each glycopeptide, the number of samples in which the feature is detected (HCC vs CIRR), MELD-adjusted *p*-value, and fold change (FC) are provided.

The 11 glycopeptides are reported as a discovery-stage candidate set of individual intact glycopeptide features and do not represent a locked composite biomarker model with a defined scoring rule or clinical cutoff. The dot plots of representative candidates showed consistent differences in normalized intensity between CIRR and HCC (Figure 4). Among the N-linked markers, *CFP*, and *AHSG* were shifted toward lower intensities in HCC (*p* = 3.9 × 10^–9^ and *p* = 1.4 × 10^–8^, respectively; Table 2). For the O-linked markers, IGH c1218 heavy, IGHV3–48, and IGHD2–2 showed higher intensity in HCC (Figure 4d), whereas IGH c3277 showed lower intensity in HCC. The separation observed in these distributions matched the corresponding fold-change directions reported for the prioritized features (Table 2). Representative annotated HCD MS/MS spectra of these selected N-linked and O-linked intact glycopeptides are provided in Figures S3 and S4, respectively.

**Figure 4:**
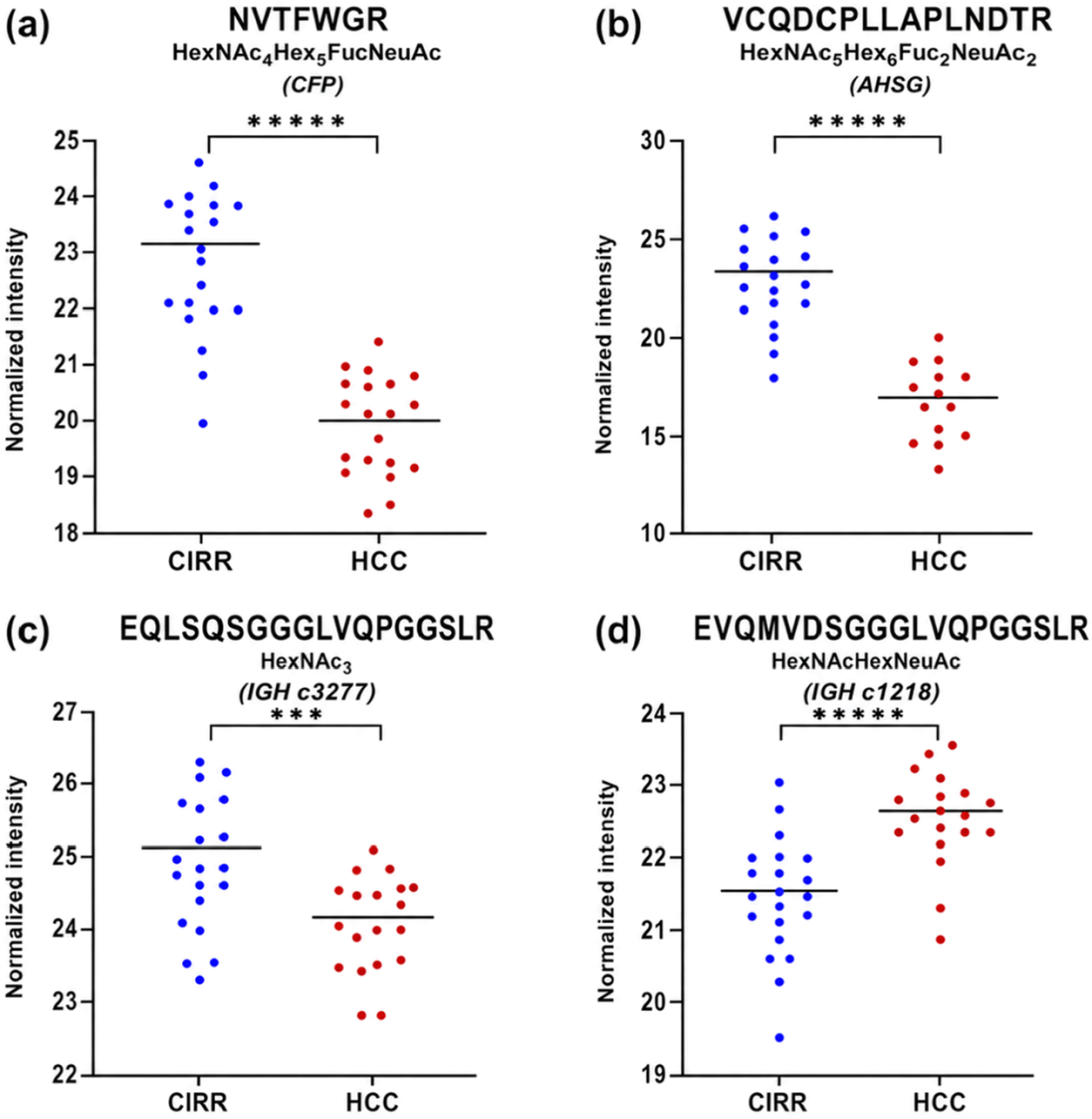
Intact glycopeptide-level abundance differences for representative candidate features distinguishing HCC from cirrhosis.

Univariate ROC analysis was used to assess the ability of selected glycopeptide markers to discriminate HCC from cirrhosis and to benchmark performance against clinically measured AFP (AUC = 0.664) (Figure 5). The glycopeptide markers showed higher discriminatory performance than AFP, with AUC values ranging from 0.786 to 0.990. The highest AUCs were observed for *AHSG* (AUC = 0.990) and *CFP* (AUC = 0.970), followed by *IGH c1218* (AUC = 0.865) and *IGHc 3277* (AUC = 0.786) (Figure 5).

**Figure 5:**
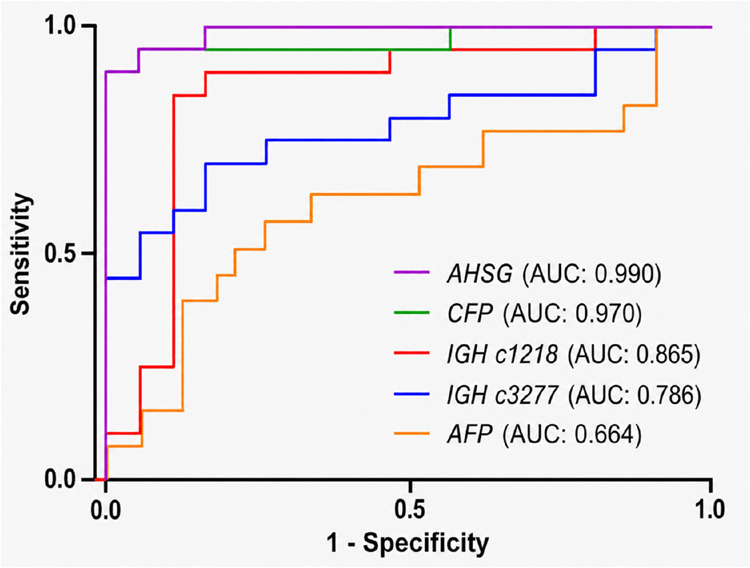
Receiver Operating Characteristic (ROC) curves demonstrating the diagnostic performance of the intact glycopeptide candidate markers and AFP in discriminating HCC from CIRR.

Protein–protein interaction (PPI) and pathway enrichment analyses were performed using the proteins corresponding to the prioritized glycopeptide features (Table 2) to provide biological context for the prioritized markers (Figure 6). The interaction network formed a connected module enriched for complement- and inflammation-associated proteins, including nodes linked to the terminal complement complex (C8/C9) and complement-associated factors (e.g., CFP), along with additional circulating proteins represented in the panel Figure 6a. Pathway enrichment highlighted immune and innate-response themes among the top signals, including neutrophil degranulation and the complement cascade, and also identified enrichment for pathways related to glycosylation and lipid metabolism (e.g., O-linked glycosylation and sphingolipid metabolism), as well as nuclear receptor–associated processes such as LXR/RXR activation and androgen signaling (Figure 6b). The full ranked enrichment results and plots are provided in the Supporting Information (Figure S5).

**Figure 6:**
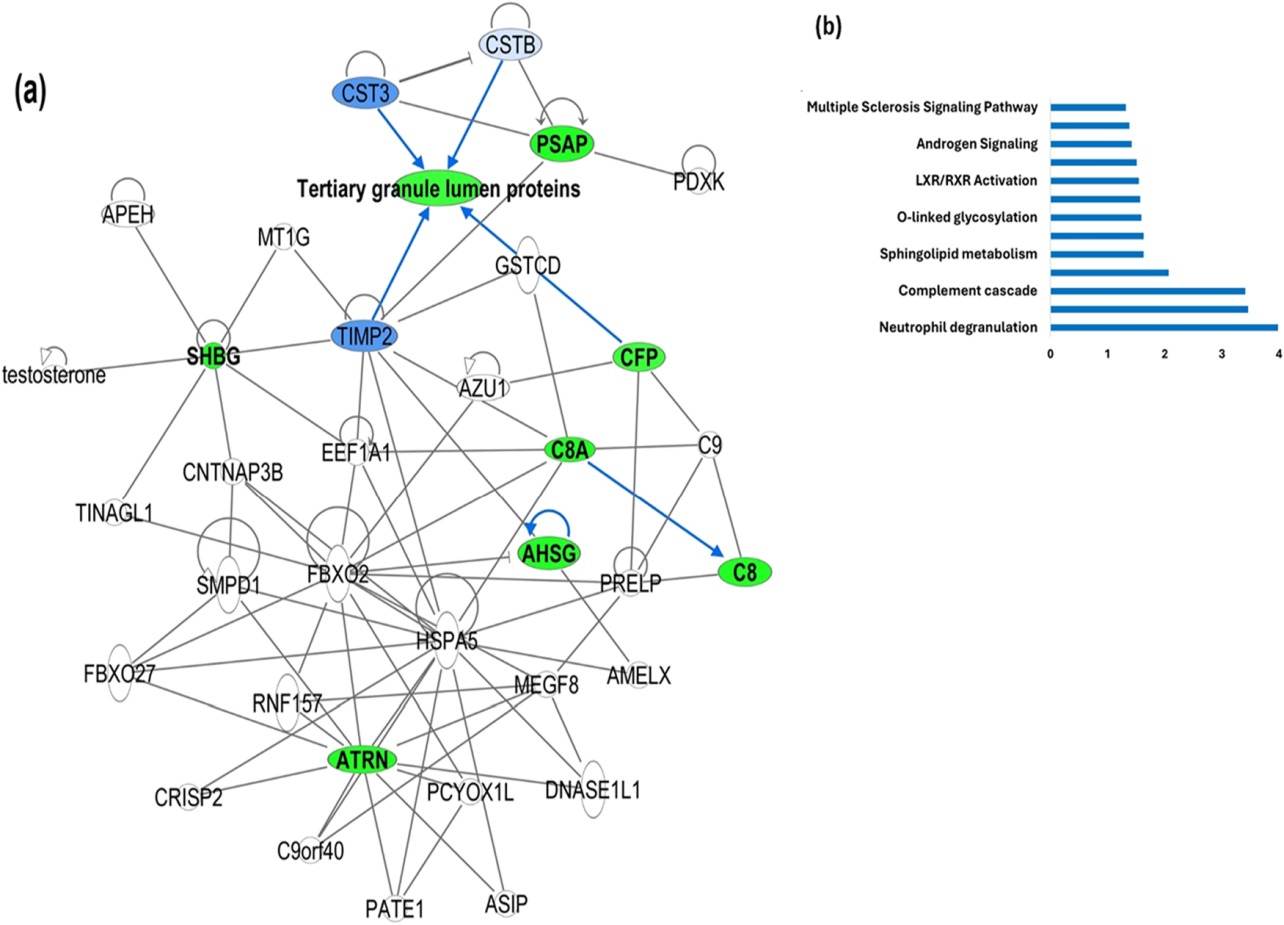
IPA analysis (a) Protein–protein interaction (PPI) network (b) Top ten canonical pathways enriched with differentially expressed precursor proteins.

## Discussion

### Workflow Significance

This study integrates parallel profiling of N-linked and O-linked intact glycopeptides with a multialgorithm feature-selection strategy to prioritize circulating candidates that distinguish HCC from cirrhosis in a clinically relevant serum setting. The analytical foundation for comparative modeling was supported by stable longitudinal QC behavior and chromatographic reproducibility across repeated QC injections, consistent with the level of stability required for discovery-phase biomarker studies in complex biofluids. Beyond technical performance, the breadth of glycoproteome coverage across both glycosylation classes reinforces the practical value of paired N/O measurements. Many circulating proteins can carry both N- and O-glycosylation, yet detectable sites and glycoforms can differ substantially between classes, and integrated profiling can therefore expand measurable disease-associated signals beyond either modality alone. This approach is well aligned with prior work emphasizing that liver disease and HCC involve glycosylation remodeling and that site- and glycoform-resolved measurements can add information beyond bulk protein abundance or glycan-only readouts.^31^

### Biological Insight of Prioritized Candidates

A key design element was combining prevalence-aware filtering (≥70% presence for the primary quantitative matrix) with statistical rescue of low-prevalence features (<30% presence evaluated by chi-square), followed by consensus prioritization across multiple feature-selection approaches. This is particularly relevant in circulating glycoproteomics, where informative biology can appear either as abundance shifts among broadly observed glycopeptides or as strong appearance/disappearance patterns driven by disease state, detection limits, or changes in glycoform occupancy. Requiring candidates to recur across multiple selection methods biases the shortlist toward signals that are robust to model choice and reduces reliance on any single algorithm’s inductive bias.

Within the prioritized features, three candidates are especially promising for follow-up: ATRN, CFP, and the O-glycopeptide feature assigned to *IGH c3277* because they captured complementary discriminatory structure and map to biologically plausible themes for the cirrhosis-to-HCC transition. The ATRN-associated glycopeptide showed a pronounced group-skewed detectability pattern in the cohort, a behavior that is often favorable for translation because it is less sensitive to small scaling differences than modest fold changes. *ARTN* is a circulating glycoprotein rapidly upregulated on activated T cells and released extracellularly; mechanistic studies report that it mediates T cell–monocyte interactions and can promote monocyte adherence/spreading and immune-cell clustering.^32^ These known immune-interaction functions provide a plausible biological context for an ATRN glycoform signal emerging during immune remodeling in advanced chronic liver disease and malignant transformation.

A complementary innate immune axis is provided by CFP. *CFP* is the only known intrinsic positive regulator of the alternative complement pathway and stabilizes the C3/C5 convertases, amplifying complement activation.^33^ Complement dysregulation is increasingly recognized as relevant to cancer biology and tumor–immune interactions, and the emergence of complement-linked candidates in an unbiased glycopeptide feature-selection workflow is consistent with the concept that immune/complement remodeling contributes to clinically observable serum signatures in the HCC–CIRR setting.^34^ Finally, the *IGH c3277*-assigned O-glycopeptide was broadly detected yet shifted in abundance between groups, a pattern consistent with glycoform remodeling and/or site occupancy effects rather than a simple gain/loss of the underlying protein. This interpretation aligns with the broader literature, indicating that disease-associated glycosylation remodeling captured at the intact glycopeptide level can stratify liver disease states and may reflect biology not observable with protein-total measurements alone.^35^ The three candidates displayed different degrees of detected glycoform diversity. *CFP* is represented by a single prioritized N-linked glycopeptide glycoform, whereas the other highlighted proteins show evidence of multiple glycosylation sites and diverse glycan compositions. This distinction is important because the discriminatory signal may be driven by a specific peptide glycan feature for some proteins, while for others it may reflect broader site- and glycoform-level heterogeneity. These observations further support the value of intact glycopeptide-level analysis for resolving disease-associated molecular changes that would be obscured by protein-level measurements alone.

### Interpretation of Glycopeptide-Level Signals

The candidate markers were selected based on intact glycopeptide-level intensities; therefore, each feature reflects a combined signal from the peptide backbone, glycosylation site, and attached glycan. Thus, abundance differences may arise from total protein abundance, glycosite occupancy, glycan composition, and/or site-specific glycosylation remodeling. We therefore interpret these candidates as discriminative glycopeptide-level features rather than protein-independent glycosylation markers. Importantly, intact glycopeptide analysis preserves site- and glycoform-specific information that is not captured by conventional protein-level measurements.

### Clinical Context

A practical motivation for developing intact-glycopeptide biomarkers in the HCC–CIRR setting is the recognized limitation of AFP as a stand-alone surveillance or diagnostic discriminator. AFP performance varies across cohorts and thresholds, and sensitivity for early stage disease can be modest; AFP-negative HCC is well documented, motivating continued development of complementary biomarker strategies.^36^ In this cohort, selected glycopeptide candidates demonstrated stronger univariate discrimination than AFP, supporting the premise that glycoform-resolved features can capture disease-associated information not reflected by AFP alone, particularly within a cirrhosis background where inflammation and regeneration complicate the interpretation of single analytes.

### Comparison with Prior HCC Glycoproteomics Studies and Rationale for Parallel N- and O-Glycoproteomics

To place our findings in the context of prior liver disease glycoproteomics studies, we compared our results with previously reported N- and O-linked glycopeptide alterations in HCC and cirrhosis. Previous glycoproteomics studies in HCC and cirrhosis have primarily focused on N-linked glycosylation, particularly on altered fucosylation, branching, and sialylation of serum/plasma glycoproteins such as haptoglobin and immunoglobulins.^37^ Site-specific N-glycopeptide studies have shown that haptoglobin glycoforms can distinguish HCC from cirrhosis, supporting the value of intact glycopeptide-level analysis for liver disease biomarker discovery.^31,38^ In comparison, O-linked glycoproteomics remains less extensively explored in HCC, although emerging studies indicate that intact O-glycopeptides, including haptoglobin-derived O-glycopeptides, may provide additional disease-relevant information. The advantage of conducting O-linked glycoproteomics in parallel with N-linked glycoproteomics is such that these approaches interrogate distinct and complementary glycosylation spaces. N-linked glycosylation is constrained by the N-X-S/T sequon. It is highly informative for secreted and membrane glycoproteins. In contrast, O-linked glycosylation occurs mainly on serine/threonine residues without a strict consensus motif and is commonly associated with mucin-like, secreted, extracellular, and cell-surface proteins. Therefore, parallel N- and O-linked glycoproteomics expands the detectable glycosylation landscape and may capture complementary features of HCC progression, including altered hepatocyte secretion, immune/inflammatory responses, extracellular matrix remodeling, and tumor-associated glycan remodeling.

### Limitations and Future Work

This discovery-scale study (*n* = 20/20) is cross-sectional; therefore, the identified glycopeptide features represent a snapshot of serum differences between established HCC and cirrhosis, not longitudinal markers of HCC progression. Larger independent and longitudinal cohorts will be needed to validate these candidates, assess generalizability across cirrhosis etiologies and disease severity, and determine whether they track disease evolution or predict malignant transformation.

## Conclusion

An integrated workflow was developed and applied for parallel N-linked and O-linked intact glycopeptide profiling in serum and coupled with a prevalence-aware, multialgorithm feature-selection strategy to discriminate HCC from cirrhosis. This approach produced a compact set of prioritized glycopeptide features best interpreted as discovery-stage candidate diagnostic markers intended to distinguish HCC from cirrhosis and require independent validation before clinical translation. Among the shortlisted markers, ATRN, CFP, *IGH c1218, and IGH c3277* emerged as priority candidates, representing immune/complement and glycoform-remodeling signals relevant to the cirrhosis-to-HCC transition. These findings provide a clear path for follow-up targeted MS validation and absolute quantification, with parallel benchmarking against orthogonal immunoassays, toward developing clinically translatable biomarkers for HCC detection in cirrhotic patients.

## Supplementary Material





## Data Availability

Data generated in this work is available via the ProtemeXchange Consortium via the PRIDE partner repository with the identifier PXD075370.

## Notes

PCA performed separately for the N-linked glycopeptides (Figure S1); PCA performed separately for the O-linked glycopeptides (Figure S2); representative annotated MS/MS spectra of N-linked intact glycopeptides (Figure S3); representative annotated MS/MS spectra of O-linked intact glycopeptides (Figure S4); functional enrichment analysis (Figure S5); top five N-linked glycopeptides ranked by feature selection methods (Table S3); top five O-linked glycopeptides ranked by feature selection methods (Table S4); top five combined glycopeptides ranked by feature selection methods (Table S5) (PDF)
N-linked glycopeptides from HCC and CIRR Groups (Table S1); O-linked glycopeptides from HCC and CIRR Groups (Table S2) (XLSX)
