# Supplementary material for: Glycoproteome Profiling of Human Serum for Hepatocellular Carcinoma Biomarker Discovery
Source: J Proteome Res. 2026 Jun 17;25(7):3705–15. doi: 10.1021/acs.jproteome.6c00258 (PMC13339751; doi:10.1021/acs.jproteome.6c00258)
Supplement: Supplementary file 1 [file pr6c00258_si_001.pdf]

Glycoproteome Profiling of Human Serum for Hepatocellular Carcinoma Biomarker Discovery

Muhammad Salman Sajid[1], Rency S. Varghese[1], Shafaq Saleem[1], Xinran Zhang[1], Alexander Kroemer,[2] Habtom W. Ressom[1]*

[1]Department of Oncology, Lombardi Comprehensive Cancer Center, Georgetown University Medical Center, Washington, DC 20057, USA

[2]MedStar Georgetown Transplant Institute, MedStar Georgetown University Hospital, and the Center for Translational Transplant Medicine, Georgetown University Medical Center, Washington, DC 20057, USA

*Corresponding Author
Prof. Habtom W. Ressom
Department of Oncology
Lombardi Comprehensive Cancer Center
Georgetown University Medical Center
Washington, DC 20057, USA
Email: hwr@georgetown.edu

Table of Content

Figure S1: PCA performed separately for the N-linked glycopeptides S3

Figure S2: PCA performed separately for the O-linked glycopeptides S3

Figure S3: Representative annotated MS/MS spectra of N-linked intact glycopeptides S4

Figure S4: Representative annotated MS/MS spectra of O-linked intact glycopeptides S5

Figure S5: Functional enrichment analysis S6

Table S1: Table S1: N-linked glycopeptides from HCC and CIRR Groups (XLSX)

Table S2: Table S2: O-linked glycopeptides from HCC and CIRR Groups (XLSX)

Table S3: Top five N-Linked glycopeptides ranked by feature selection methods S7

Table S4: Top five O-Linked glycopeptides ranked by feature selection methods S8

Table S5: Top five combined glycopeptides ranked by feature selection methods S9

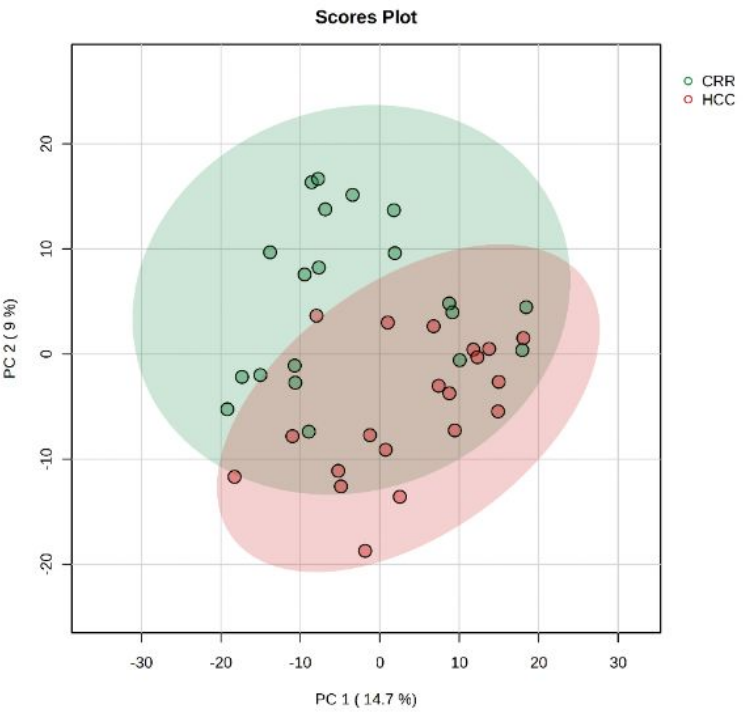

Figure S1: PCA performed separately for the N-linked glycopeptides

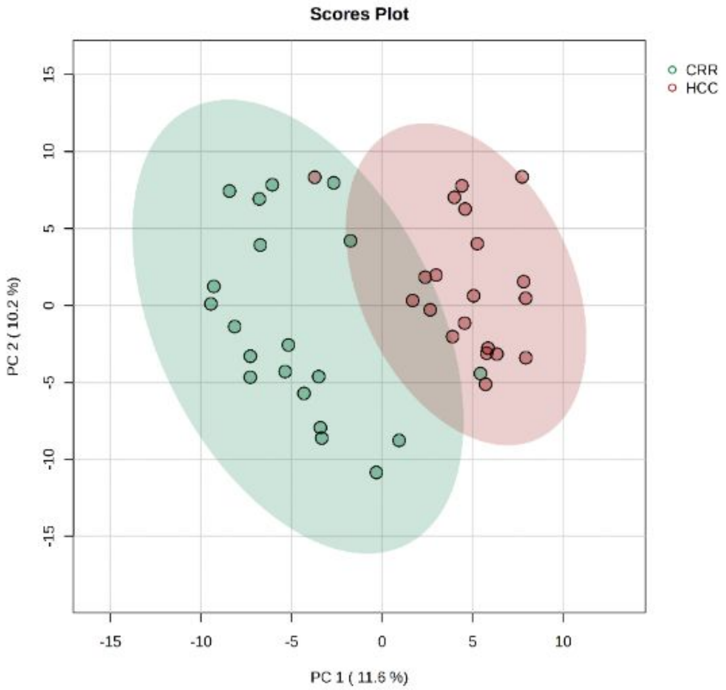

Figure S2: PCA performed separately for the O-linked glycopeptides.

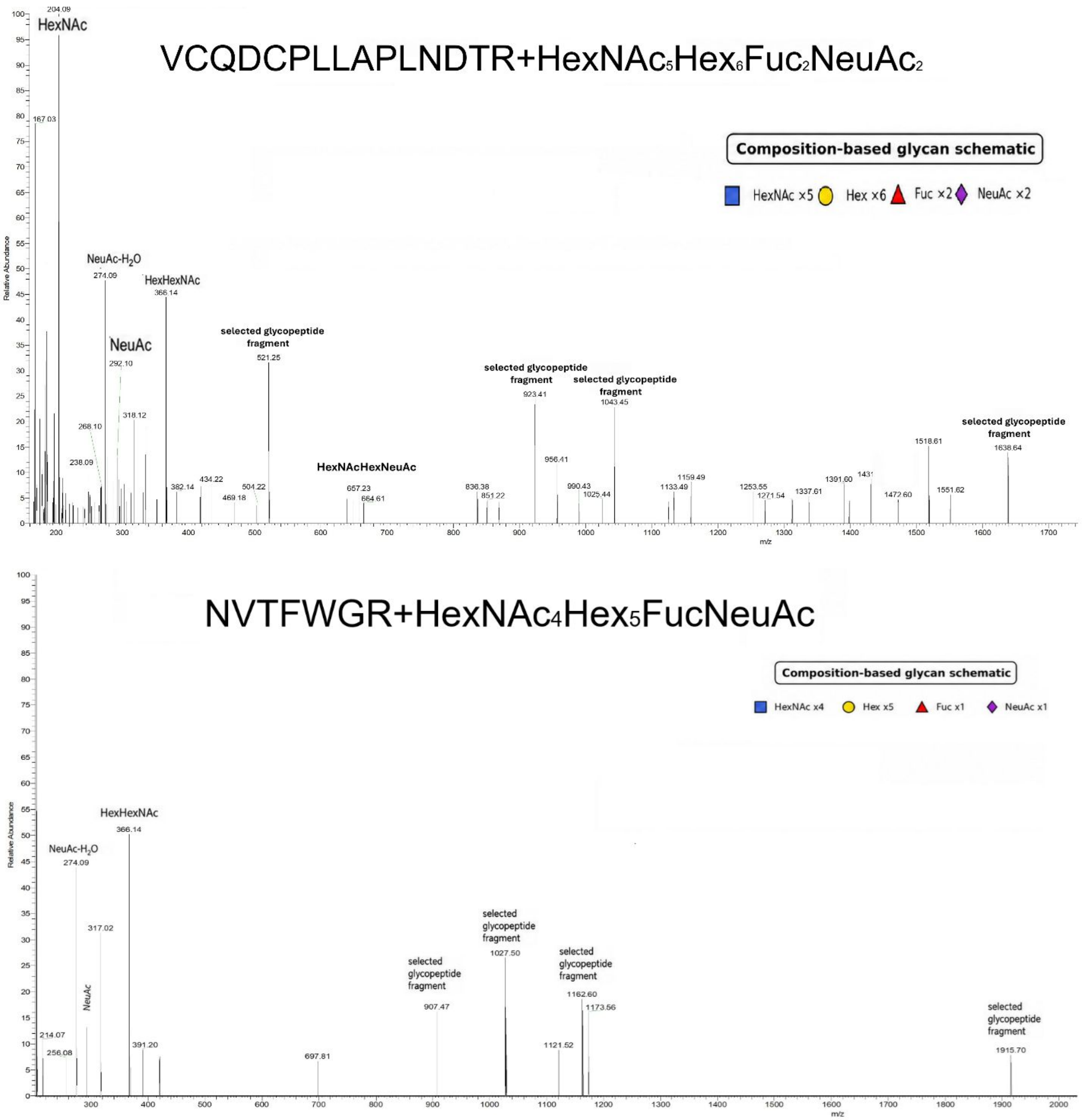

Figure S3: Representative annotated MS/MS spectra of N-linked intact glycopeptides.

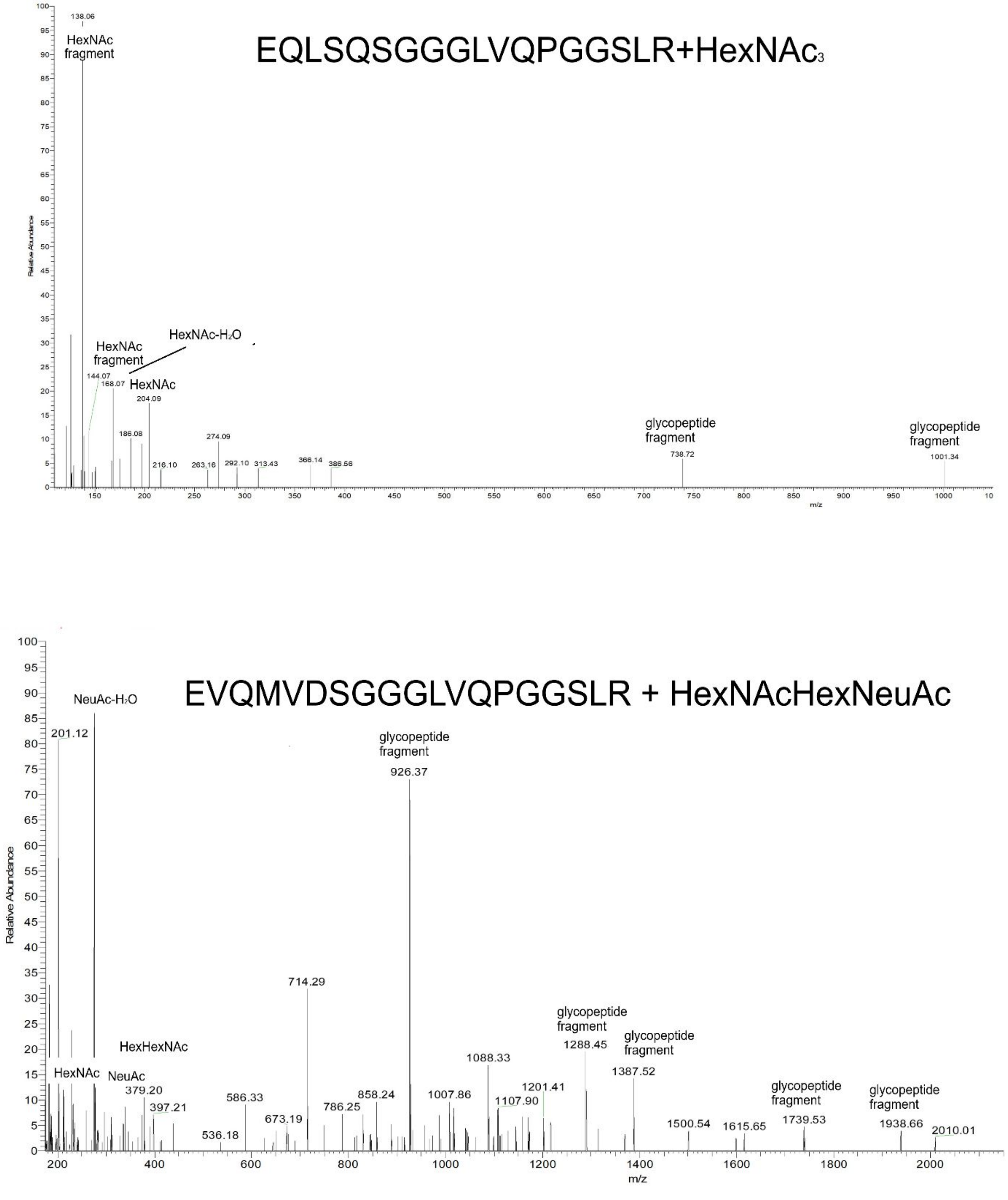

Figure S4: Representative annotated MS/MS spectra of O-linked intact glycopeptides.

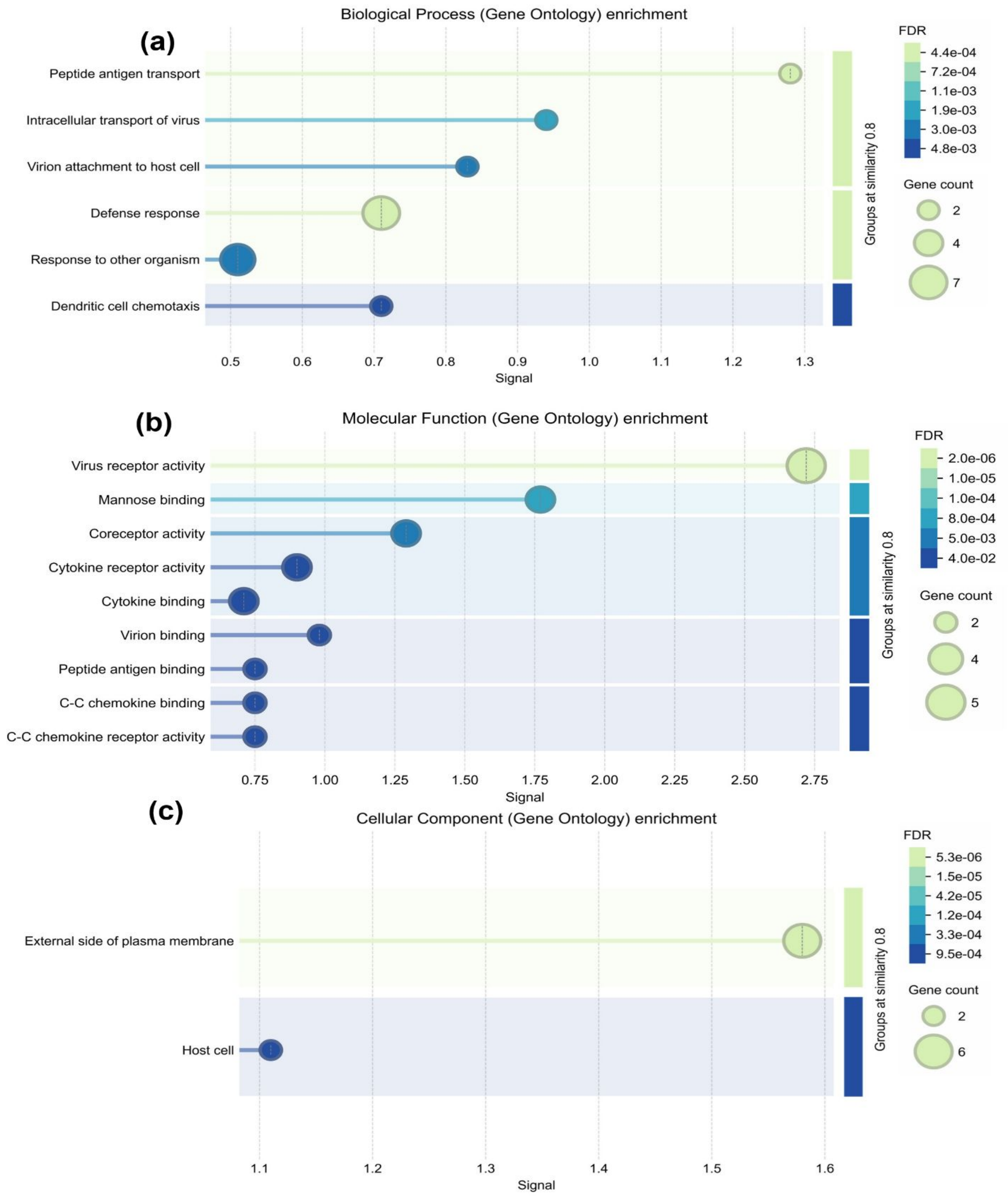

Figure S5: Functional enrichment analysis. Gene ontology (GO) enrichment analysis (a) highlighting biological processes (BP), (b) molecular functions (MF), and (c) cellular components (CC) associated with DEPs.

Table S3. N-Linked glycopeptides ranked in the top five by more than one feature selection method.

Protein	Sequence	Glycan	MH+ [Da]	Detected in CIRR	Detected in HCC	SelectKBest	SVM-RFE	ElasticNet	Transformer- RFE	RF
ATRN	VFHIHNESWVLLTPK	$HexNAc_2$ Hex_4	2874.35	19	0		✔			✔
F11	VYSGILNQSEIK	$HexNAc_4$ Hex_5 NeuAc	3264.40	16	1	✔				
APOB	FNSSYLQGTNQITGR	$HexNAc_4$ Hex_5	3308.40	17	20				✔	
CFP	NVTFWGR	$HexNAc_4$ Hex_5 Fuc NeuAc	2939.18	20	20	✔	✔			
LUM	LHINHNNLTESVGPLPK	$HexNAc_4$ Hex_5 Fuc NeuAc	3942.74	20	20	✔				
C8A	GGSSGWSGGLAQNR	$HexNAc_4$ Hex_5 $NeuAc_2$	3538.39	20	2	✔	✔			✔
SHBG	SHEIWTHSCPQSPGNGTDASH	$HexNAc_4$ Hex_5 NeuAc	4218.64	19	3	✔	✔	✔	✔	✔
AHSG	VCQDCPLLAPLNDTR	$HexNAc_5$ Hex_6 Fuc_2 $NeuAc_2$	4633.86	20	11		✔			✔
FLJ00385	EEQFNSTFR	$HexNAc_4$ Hex_3	2455.99	10	0			✔		
AFM	FNETTEK	$HexNAc_5$ Hex_6 Fuc $NeuAc_2$	3584.36	3	13			✔		
HPX	ALPQPQNVTSLLGCTH	$HexNAc_4$ Hex_5	3358.46	10	19			✔		
C4A	GLNVTLSSTGR	$HexNAc_4$ Hex_5 Fuc	2914.26	17	5			✔		
F13B	EHETCLAPELYNGNYSTTQK	$HexNAc_4$ Hex_5 Fuc_2 NeuAc	4560.84	14	17				✔	
CFP	NVTFWGR	$HexNAc_4$ Hex_5 Fuc NeuAc	2939.18	20	20				✔	
SERPINA1	YLGNATAIFFLPDEGK	$HexNAc_4$ Hex_5 NeuAc	3669.57	20	15				✔	
APOB	TIHDLHLFIENIDFNK	$HexNAc_4$ Hex_5 $NeuAc_2$	4173.79	18	4					✔
					**Accuracy**	0.900	0.925	0.900	8.875	0.900
					**AUC**	0.990	1.000	0.928	0.968	1.000

Table S4. O-linked glycopeptides ranked in the top five by more than one feature selection method.

Protein	Sequence	Glycan	Detected in CIRR	Detected in HCC	SelectKBest	SVM-RFE	ElasticNet	Transformer-RFE	RF
IGH c3277	EQLSQSGGGLVQPGGSLR	HexNAc$_3$	20	20	✔		✔		
C-X3-C	AQDGGPVGTELFR	HexNAc Hex1NeuAc$_2$	15	1	✔	✔	✔	✔	✔
IGH c1218	EVQMVDSGGGLVQPGGSLR	HexNAc Hex NeuAc	20	15	✔	✔	✔	✔	✔
SERPINA1	DTEEEDFHVDQATTVK	HexNAc$_2$Hex$_2$NeuAc	1	10	✔				
PSAP	TNSTFVQALVEHVK	HexNAc$_2$ Hex$_2$ Fuc	17	2	✔			✔	
IG c657	QDGSEK	HexNAc$_2$ Hex Fuc NeuAc	20	2		✔	✔	✔	✔
cDNA FLJ51742	TEETTMTTQTPAPIQAPSA ILPLPGQSVER	HexNAc$_4$Hex	19	1		✔	✔		✔
KNG1	TEHLASSSEDSTTPSAQTQEK	HexNAc Hex	20	20		✔			
FN1	DQCIVDDITYNVNDTFHK	HexNAc	4	3				✔	
KNG1	DIPTNSPELEETLTHTITK	HexNAc Hex NeuAc	15	0					✔
				**Accuracy**	0.925	0.975	0.925	0.950	0.975
				**AUC**	0.993	0.998	0.993	0.998	0.998

Table S5: Combined N- and O-linked glycopeptides ranked in the top five by more than one feature selection method.

Protein	Sequence	Modifications	MH+ [Da]	Detected in CIRR	Detected in HCC	SelectK Best	SVM-RFE	ElasticNet	Transformer-RFE	RF
ATRN	VFHIHNESWVLLTPK	$HexNAc_2$ Hex_4	2874.35	19	0		✔			✔
C8A	GGSSGWSGGLAQNR	$HexNAc_4$ Hex_5 $NeuAc_2$	3538.39	20	2		✔			✔
AHSG	VCQDCPLLAPLNDTR	$HexNAc_5$ Hex_6 Fuc_2 $NeuAc_2$	4633.86	20	11		✔			
KNG1	ITYSIVQTNCSK	$HexNAc_5$ Hex_6 Fuc $NeuAc_2$	4129.66	20	19				✔	
ZINC-ALPHA-GP	FGCEIENNR	$HexNAc_4$ Hex_5 Fuc $NeuAc_2$	3489.32	19	20				✔	
APOD	ADGTVNQIEGEATPVNLTEPAK	$HexNAc_4$ Hex_5 $NeuAc_2$	4458.89	20	11				✔	
SHBG	SHEIWTHSCPQSPGNGTDASH	$HexNAc_4$ Hex_5 NeuAc	4218.64	19	3					✔
HPX	ALPQPQNVTSLLGCTH	$HexNAc_5$ Hex_6 Fuc_3 $NeuAc_2$	4743.95	17	1					✔
PSAP	TNSTFVQALVEHVK	$HexNAc_2$ Hex_2 Fuc	2449.16	17	2	✔				
IGH c1218	EVQMVDSGGGLVQPGGSLR	HexNAc Hex NeuAc	2542.17	20	15	✔		✔		
PRG4	VTTPDTSTTQHNK	$HexNAc_2$ Hex_2 $NeuAc_2$	2742.14	1	1	✔	✔			
IGH C3545	VQMVESGGGLVQPGGSLR	$HexNAc_3$ Hex	2542.20	17	15	✔				
cDNA FLJ517442	TEETTMTTQTPAPIQAPSA ILPLPGQSVER	$HexNAc_4$ Hex	4138.98	19	1	✔	✔	✔		
IGH c3277	EQLSQSGGGLVQPGGSLR	$HexNAc_3$	2379.15	20	20			✔		
KNG1	TEHLASSSEDSTTPSAQTQEK	HexNAc Hex	2599.13	20	20			✔		
C-X3-C motif	AQDGGPVGTELFR	HexNAc Hex $NeuAc_2$	2293.99	15	1			✔		
PRG4	SPDESTPELSAEPTPK	$HexNAc_2$ Hex2 $NeuAc_2$	2997.24	20	20				✔	
IGH c1218	VQMVDSGGGLVQPGGSLR	HexNAc Hex $NeuAc_2$	2704.22	20	19				✔	
IG c657	QDGSEK	$HexNAc_2$ Hex Fuc NeuAc	1668.65	20	2					✔
				**Accuracy**		0.925	0.925	0.975	0.900	0.875
				**AUC**		0.993	1.000	0.995	0.908	1.000
